# Special Issue “Molecules at Play in Neurological Diseases”

**DOI:** 10.3390/cimb47080600

**Published:** 2025-07-30

**Authors:** Dumitru Andrei Iacobas

**Affiliations:** 1 Personalized Genomics Laboratory, Undergraduate Medical Academy, School of Public and Allied Health, Prairie View A&M University, Prairie View, TX 77446, USA; daiacobas@pvamu.edu; 2 DP Purpura Department of Neuroscience, Albert Einstein College of Medicine, Bronx, New York, NY 10461, USA

Pending the approval of the Institutional Review Boards (IRBs), researchers can access clinicians’ tools to explore the molecular phenomena affected by neurological disorders in living humans. Among others, they can use various imaging techniques (e.g., [1]) and also could analyze blood (e.g., [2,3]), bodily waste (urine and feces, e.g., [4]) or surgically removed tissues (e.g., [5]). However, testing novel therapeutic avenues on living humans faces drastic legal, moral and religious constrains beyond the ethics rules established by the Declaration of Helsinki [6] and needs a long chain of approvals before being accepted for clinical trials. Therefore, frequent choices are use of cell cultures isolated from surgically removed tissues of diseased persons (e.g., [7]) or replication of the disorder’s major symptoms on animal models (e.g., [8,9,10]). An intermediate choice is use of genetically engineered animal precursor cell line (e.g., [11]).

Both cell cultures and animal models were instrumental for our incremental understanding of nervous system pathology and it is hard to imagine what level of knowledge and medicine we would have today without them. Particularly, primary cell cultures have been essential tools for investigating the roles of various cell phenotypes in brain normal and disease states. For example, many key functions of central glial cells in metabolism and intercellular calcium signaling were uncovered through tissue culture methods, and most of these findings were later validated in vivo (e.g., [12]). Animal models were also indispensable systems for exploring novel therapeutic avenues for infantile spasms (e.g., [13]), and many other severe pathologies. However, neither cell cultures, nor animal models can perfectly reproduce the complexity of the human nervous system and one needs to take into account and finds ways to overcome their limitations. This Editorial summarizes part of the challenges experienced when investigating neurological disorders in cell lines purchased from authorized vendors or in (genetically, chemically, or mechanically) engineered animal models.

The four major aspects one has to consider when using standard cell lines to study the neurological disorders are:

(1) The cell lines were formed by culturing cells collected either from cancer tumors of the nervous system (like neuroblastoma, astrocytoma, lymphoma, brain metastases of prostate adenocarcinoma etc.) or immortalized for better preservation after collection from an affected (but not cancerous) tissues. In all cases, the cell cycle was considerably modified, so that translating the results into what might happen inside the real tissue of the living person is questionable owing to a totally different dynamics and outputs of the biological processes (e.g., [14]).
(2) In a monoculture, the cells are missing the normal hetero-cellular environment from the tissue that strongly affects all the inner molecular processes. In previous studies, we reported that the transcriptomes of astrocytes and oligodendrocytes were substantially different when profiled in separate cultures than when profiled co-cultured in insert systems [15,16], sharing the same medium even without physically touching each-other. The differences were not only in the expression levels of the genes but also in the strength of the homeostatic control of the transcripts’ abundances and in the gene networking, indicating profound remodeling of the functional pathways. This limitation of the monocultures got a partial solution through the development of the very promising technology of constructing human brain organoids (e.g., [17,18]).
(3) In addition to race, sex and age, the concrete manifestation of a disease depends on the never repeatable combination of the personal characteristics of the patients that includes but is not limited to the medical history, diet, exposure to stress and toxins, climate etc. Therefore, the donor of the selected cell line should match as many as possible characteristics of the studied person or of the homogeneous population.
(4) Any genetic manipulation of the sequence, 3d spatial configuration or expression level of a gene has ripple effects on hundreds other genes, presumably because of their integration in functional pathways. Owing to the uniqueness of the local conditions, the combination and the amplitudes of all other affected genes is never repeatable. Moreover, about 1/1000 of the nucleotides are randomly mutated just because of the stochastic nature of the chemical reactions involved in the DNA replication, making difficult to blame solely the targeted gene for the observed phenotype. Sometimes, the manipulated gene is just one out of several other potential triggers of cascades of similar molecular mechanisms.

Despite being limited by the welfare rules included in the Declaration of Helsinki and subjected to protocol approval by the Institutional Animal Care and Use Committee, an animal model still allows otherwise never acceptable investigations on humans.

However, animal modelling have many challenges too. Like humans, animal features depend on species, strain, sex, age/developmental stage and hormonal status, exposure to oxygen deprivation and toxins, diet, external stimuli, medical history, and treatment (e.g., [19,20]). Importantly, the subcellular localization of certain proteins not only differs between sexes but also changes during the estrogen cycle [21], making the female animal models much more difficult to manage and interpret than their male counterparts. Therefore, choosing the right animal model is far from an easy task. Furthermore, sanitary precautions and housing conditions (e.g.,: distribution of cages and vicinity of other caged males, diet chow and water abundance and quality, environmental temperature, humidity, day and night lighting cycles) are also major modulators of the experimental results.

Nevertheless, the molecular characteristics of the model are not uniform across different regions of the organ and are affected by tissue hetero-cellular organization. We have profiled the transcriptomes of various regions of the brain, spinal cord and retina. The tissues were collected from wildtype and in-house engineered mice, rats and rabbits that modeled neuroblastoma, glioma, autism, epilepsy, glaucoma, intraventricular hemorrhage, infantile spasms, multiple sclerosis, neuroblastoma, neuropsychiatric lupus erythematosus, Charcot–Marie–Tooth disease, and oculodentodigital dysplasia. In rat experiments, we found substantial transcriptomic differences among the hippocampal dentate gyrus, CA1, CA2 and CA3 regions, as well as between the hypothalamic arcuate and periventricular nuclei. Therefore it is very important to select the right region to study and also to have a very skilled neuroanatomist in the team.

It is a widespread belief that many diseases are caused by altered sequence or/and expression level of certain critical gene(s), termed (gene) biomarker(s) (e.g., [22]). Most genomists assumed that, by engineering the same alteration in the genome of an animal, one can reproduce the key features of human disease. On this line, we induced some diseases by manipulating genes like *Gja1*, *Gjb1*, *Gjd2* that encode the connexins forming the gap junction channels in astrocytes, oligodendrocytes and neurons, and the *Fas* gene, whose loss of function mutation leads to a systemic lupus-like phenotype. In all these engineered mice, we found hundreds other genes as significantly regulated and many major functional pathways remodeled, whose alterations, dependent on the type of manipulation, were different between strains, sexes, hormonal status, and age groups. Even informative for that particular animal model, translating the results to humans should remain in hypothetical qualitative terms.

Other animal models might be acquired through chemical or mechanical treatment. For instance, glaucoma was derived in rats by crushing the optic nerve. Infantile Spasms were induced in prenatally betamethasone primed rats with NMDA, status epilepticus was induced in rats with intraperitoneal injection of kainic acid, while multiple sclerosis symptoms were satisfactorily replicated in mouse adoptive transfer experimental autoimmune encephalomyelitis obtained by injecting myelin basic protein (Mbp). Intraventricular hemorrhage was triggered in rabbit premature pups by intraperitoneal injection with 50% glycerol. However, although these animal models mimicked satisfactorily the symptoms of the corresponding human disorders, their occurrence and dynamics were different, requiring careful interpretation of the results.

In summary, albeit very useful to get an idea about the molecular phenomena that might be responsible for the development of neurological disorders, one should consider the limitations of standard cell lines and animal models to reproduce the phenomena in the human nervous system.
